# Genome-wide association study identified candidate genes controlling continuous storage root formation and bulking in hexaploid sweetpotato

**DOI:** 10.1186/s12870-019-2217-9

**Published:** 2020-01-02

**Authors:** Astère Bararyenya, Bode A. Olukolu, Phinehas Tukamuhabwa, Wolfgang J. Grüneberg, Wellington Ekaya, Jan Low, Mildred Ochwo-Ssemakula, Thomas L. Odong, Herbert Talwana, Arfang Badji, Martina Kyalo, Yao Nasser, Dorcus Gemenet, Mercy Kitavi, Robert O. M. Mwanga

**Affiliations:** 1 Department of Agricultural Production, College of Agricultural and Environmental Sciences, Makerere University, P.O. Box 7062, Kampala, Uganda; 2 Institut des Sciences Agronomiques du Burundi, Avenue de la Cathédrale – B.P. 795, Bujumbura, Burundi; 3 Department of Entomology and Plant Pathology, University of Tennessee, Knoxville, TN 37996-4560 USA; 4 International Potato Center (CIP), Avenida La Molina 1895, La Molina Apartado Postal, 1558 Lima, Peru; 5 International Livestock Research Institute, ILRI Campus, Naivasha Rd, Nairobi, 30709-00100 Kenya; 6 International Potato Center (CIP), Regional office sub-Sahara Africa, P.O. Box 25171-00603, Nairobi, Kenya; 7 International Potato Center (CIP), Plot 47, Ntinda II Road, P.O. Box 22274, Kampala, Uganda

**Keywords:** DArTseq, Genotyping-by-sequencing, GWAS, CSRFAB, GBSapp, Polyploid, SNPs, Sweetpotato

## Abstract

**Background:**

Continuous storage root formation and bulking (CSRFAB) in sweetpotato is an important trait from agronomic and biological perspectives. Information about the molecular mechanisms underlying CSRFAB traits is lacking.

**Results:**

Here, as a first step toward understanding the genetic basis of CSRFAB in sweetpotato, we performed a genome-wide association study (GWAS) using phenotypic data from four distinct developmental stages and 33,068 single nucleotide polymorphism (SNP) and insertion-deletion (indel) markers. Based on Bonferroni threshold (*p*-value < 5 × 10^− 7^), we identified 34 unique SNPs that were significantly associated with the complex trait of CSRFAB at 150 days after planting (DAP) and seven unique SNPs associated with discontinuous storage root formation and bulking (DCSRFAB) at 90 DAP. Importantly, most of the loci associated with these identified SNPs were located within genomic regions (using *Ipomoea trifida* reference genome) previously reported for quantitative trait loci (QTL) controlling similar traits. Based on these trait-associated SNPs, 12 and seven candidate genes were respectively annotated for CSRFAB and DCSRFAB traits. Congruent with the contrasting and inverse relationship between discontinuous and continuous storage root formation and bulking, a DCSRFAB-associated candidate gene regulates redox signaling, involved in auxin-mediated lateral root formation, while CSRFAB is enriched for genes controlling growth and senescence.

**Conclusion:**

Candidate genes identified in this study have potential roles in cell wall remodeling, plant growth, senescence, stress, root development and redox signaling. These findings provide valuable insights into understanding the functional networks to develop strategies for sweetpotato yield improvement. The markers as well as candidate genes identified in this pioneering research for CSRFAB provide important genomic resources for sweetpotato and other root crops.

## Background

Perennial plants, species that live for more than 2 years, account for 13% of food crops and provide advantages over annual crops because they increase the carbon of storage organs [1] and reduce soil erosion due to their longer growing seasons [2]. While there is increasing interest in perennializing annual grains [1], sweetpotato is cultivated as an annual. It is perennial in nature with varying degrees of maturation and senescence that lead to short and long growing periods. The genetic variation underlying this trait makes it amenable to breeding sweetpotato for commercial and subsistence farming systems. Commercial farming requires synchronized maturity or discontinuous storage root formation and bulking (DCSRFAB) at harvest. Subsistence agricultural systems use piecemeal/multiple harvesting strategies to increase profitability by ensuring availability of their product over a longer growing season due to continuous storage root formation and bulking (CSRFAB). Perenniality in sweetpotato is associated with CSRFAB [3] due to its capacity to keep vegetative growth overtime that leads to increased photosynthetic activity and continuous dry matter partitioning into the storage root organ [4]. CSRFAB genotypes primarily invest in vegetative growth and later change to a reproduction phase by enhanced carbon partitioning to storage root development while continuing vegetative growth [5, 4]. This drastically increases productivity due to increased photosynthesizing green materials in CSRFAB genotypes compared to DCSRFAB genotypes [6]. Sweetpotato cultivars are capable of delaying senescence and maintaining carbon assimilation due to persistent photosynthetic activity over longer periods [7].

Sweetpotato is an important food crop in sub-Saharan Africa (SSA). Its production continues to increase in east and central Africa due to recent interests in the crop for its unsurpassed healthy promoting values and being staple food crop in the region. High production in the region is recorded in Uganda and Tanzania [8], however, based on per capita production, Rwanda, Tanzania and Burundi are the top sweetpotato consumers with 80.6, 64.1 and 56.9 kg per capita, respectively. In east African countries, piecemeal harvesting is the predominant harvesting practice among small and medium scale sweetpotato farmers. This practice consists of sequentially uprooting matured storage roots from the same sweetpotato plants on a mound, ridge or portion of the field for one or several meal(s), or for a ready market. The method is also applied in other root and tuber crops including potato [9] and cassava. While the practice keeps the storage roots in-ground and provides possibilities for continuous market along the cropping season, it also creates room for extended storage root initiation and bulking allowing increased production for the next harvest [7]. Breeding and selection have been based on one-time harvesting (DCSRFAB) and there has been no direct effort to understand the genetics underlying traits associated with these common harvesting practices in sweetpotato farming systems. Although piecemeal harvesting is recognized as important in sub-Saharan Africa (SSA), it can also be applied in temperate regions for small gardeners or for providing fundamental understanding of genetic basis for perenniality.

Through various research networks, sweetpotato breeders across countries and continents have shown outstanding consistency in the general areas of priority for breeding, most of which point to the need for increased yield potential and resistance to biotic and abiotic stresses. Frequently mentioned breeding objectives, targeting increased sweetpotato adoption and wide utilization, revolve around improvement of fresh storage root yield, storage root dry matter content (DMC), resistance to principal local pests and diseases, tolerance to adverse soil and climatic conditions, good plant habit, ornamental quality and other quality traits [10]. Considerable research is now directed to enhance traits of nutritional value (such as provitamin A carotenoid, micro-nutrients) and industrial use (such as starch). The biofortification is envisioned to overcome vitamin A deficiencies common among resource-poor peasants, while increased yields will enhance commercial production of the crop [11].

Several reports have described the anatomy and physiological processes of storage root formation and development in sweetpotato under controlled conditions [12–15] and field experiments [16–18], however, the genetic and molecular basis of CSRFAB for breeding purposes remains largely unknown. Understanding the genetic mechanisms underlying these variations, as well as the trait’s fitness are important to inform future selection practices, while taking various sweetpotato farming systems into consideration.

DNA-based genetic markers can provide great potential to assist plant breeders in the identification of genes of interest and detecting markers tightly linked to traits for the development of new cultivars. Tanaka et al. (2016) [19] reviewed molecular studies in storage root formation and identified numerous genes showing differential expression between developmental stages relative to the formation of storage roots. The formation of storage roots appears to be a default process in sweetpotato storage root development making it unclear whether a specific signal exists to initiate storage root development. Nonetheless, genetic differences should exist between sweetpotato and related plant species that do not produce storage roots. The recent whole-genome sequences of two diploid species, *Ipomoea trifida* and *I. triloba* [20], are useful resources for investigating the hexaploid sweetpotato. It is now possible to use high-density genome-wide SNP markers [21] and the robust reference genomes of the ancestral diploid progenitors [20] to understand the genetic basis of most important traits in the complex hexaploid sweetpotato genome.

In this study, we evaluated a diverse set of 358 sweetpotato breeding accessions for CSRFAB and DCSRFAB under field conditions. We genotyped this diversity panel and analyzed it for quantitative trait nucleotides (QTNs) using SNP and indel markers to identify genomic regions and polymorphisms associated with CSRFAB and DCSRFAB traits.

## Results

### Relationship between yield component traits and CSRFAB in sweetpotato

**Fig. 1:**
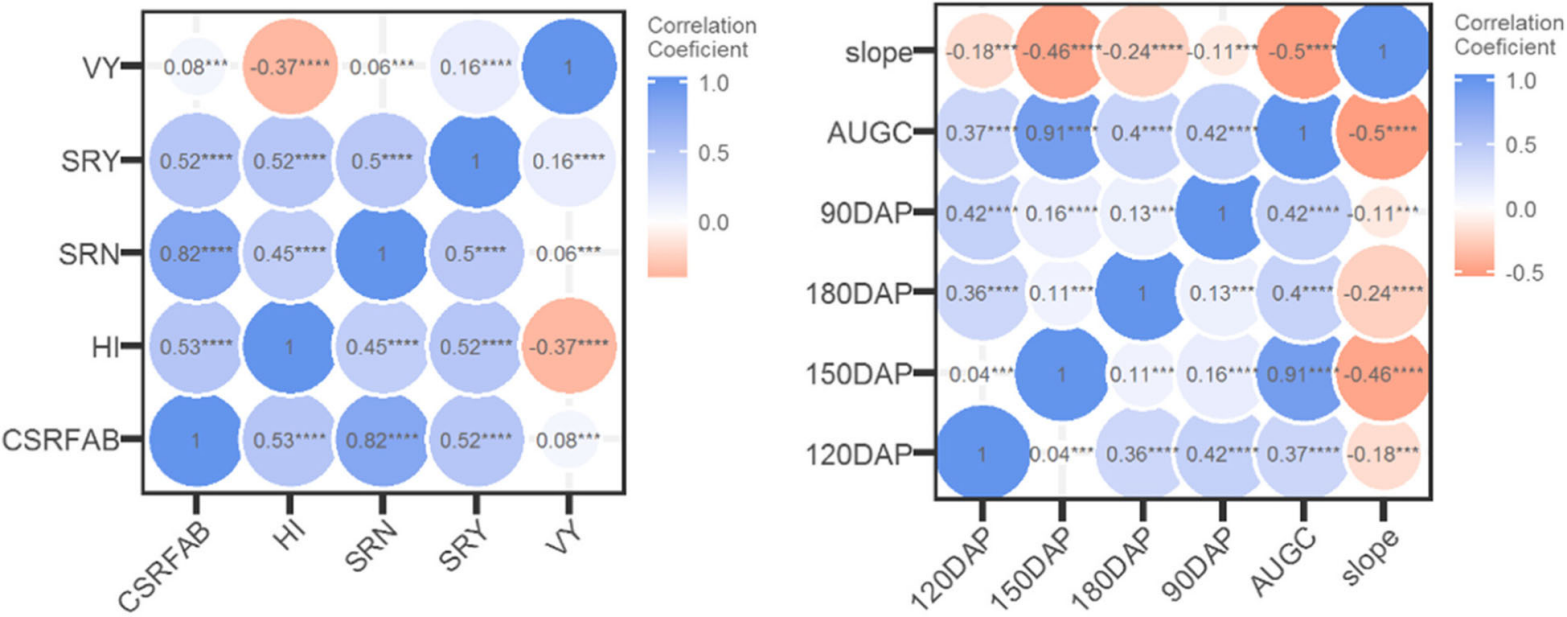
Correlation coefficients showing relationship between yield component traits (left) and CSRFAB-related traits (right). Note: CSRFAB = Continuous storage root formation and bulking; HI = harvest index; SRN = Storage root number; SRY = Storage root yield; VY = Vine yield; DAP = Days after planting; AUGPC = Area under growth progress curve

Pair-wise correlations provide information on whether two traits are related. This information about the magnitude and direction (negative or positive) of the relationship can assist in breeding selection decisions. Thus, CSRFAB was highly and positively correlated with storage root number (SRN), storage root yield (SRY) and harvest index (HI) (Fig. 1). CSRFAB responses at four harvest times, as well area under growth progress curve (AUGPC) and slope have variable correlation magnitudes (Fig. 1-right). Low to no correlations were observed among the four harvest times (90, 120, 150 and 180 DAP). We observed positive correlations between AUGPC and yield component traits at each harvest time, with the highest positive correlation coefficient recorded at 150 DAP (0.91). Negative (− 0.5) correlation coefficient between slope and AUGPC was observed. The least correlation coefficients between AUGCP and other traits were recorded at 120 DAP and 180 DAP. The slope was negatively correlated with all the traits (Fig. 1).

### Phenotypic variation in CSRFAB

**Fig. 2:**
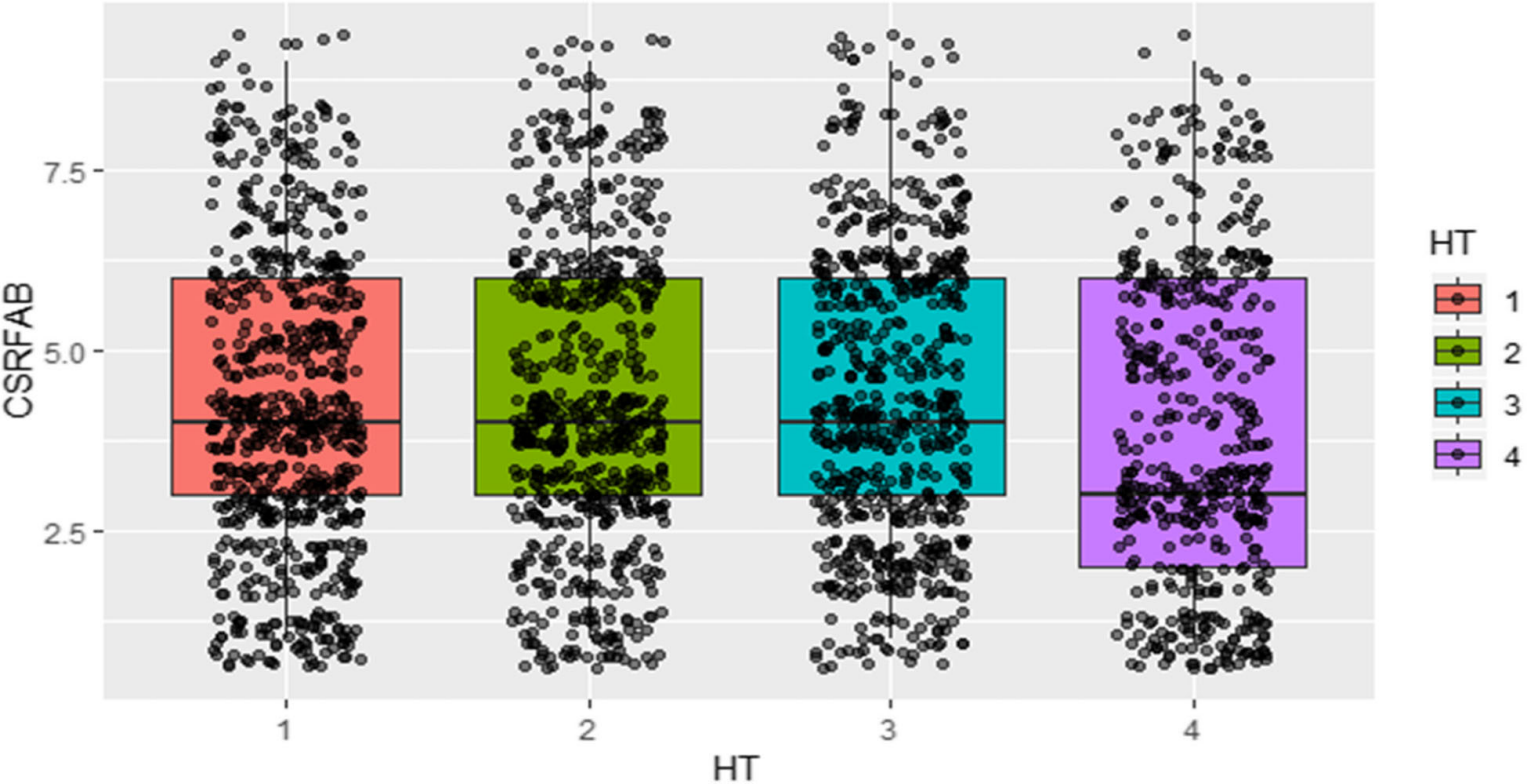
Boxplot showing overall variability and dispersion of continuous storage formation and bulking (CSRFAB) over 4 harvest times among studied cultivars in Uganda. The colors represent different harvest times (1 = 90 days after planting (DAP), 2 = 120 DAP, 3 = 150 DAP, 4 = 180 DAP, HT = harvest time) and the dot points show the distribution of genotype scores at each scoring time

The distribution of genotypic performance across the four-harvest times (HT) was visualized using a boxplot (Fig. 2). On average, across the two sites of the experiment, the mean score of CSRFAB was 3.4 with a maximum score of 7.8 and a minimum of 1.4. Our results indicate that the distribution is similar for the first three time points (90, 120 and 150 DAP) and the genotypes become much more variable at 180 DAP. The median score across the three harvest time points is about 4, while the fourth harvest time point (180 DAP) has a median score of about 3.

We previously [3] reported changes in genotypic variation over time. In DCSRFAB genotypes, genotypic variance increased as plants grew due to the increasing effect of genotypic factors in the population. The increment reached maximum at 120 DAP and decreased thereafter. Figure 2 shows a decrease in population median at harvest 4 due to high frequency of DCSRFAB genotypes that reduce storage root initiation and bulking. The drought period, which occurred between season A and season B (between 4 to 6 months after planting (MAP) can also partly explain the low population median. We observed an opposite pattern in which yield increased over time for CSRFAB due to regrowth (after 5 MAP) of CSRFAB genotypes observed after 5 months. This delayed senescence or stay green phenomena [22] resulting in high overall phenotypic variation between CSRFAB and DCSRFAB plants.

### SNP calls and allele dose-dependent genotypes

**Fig. 3:**
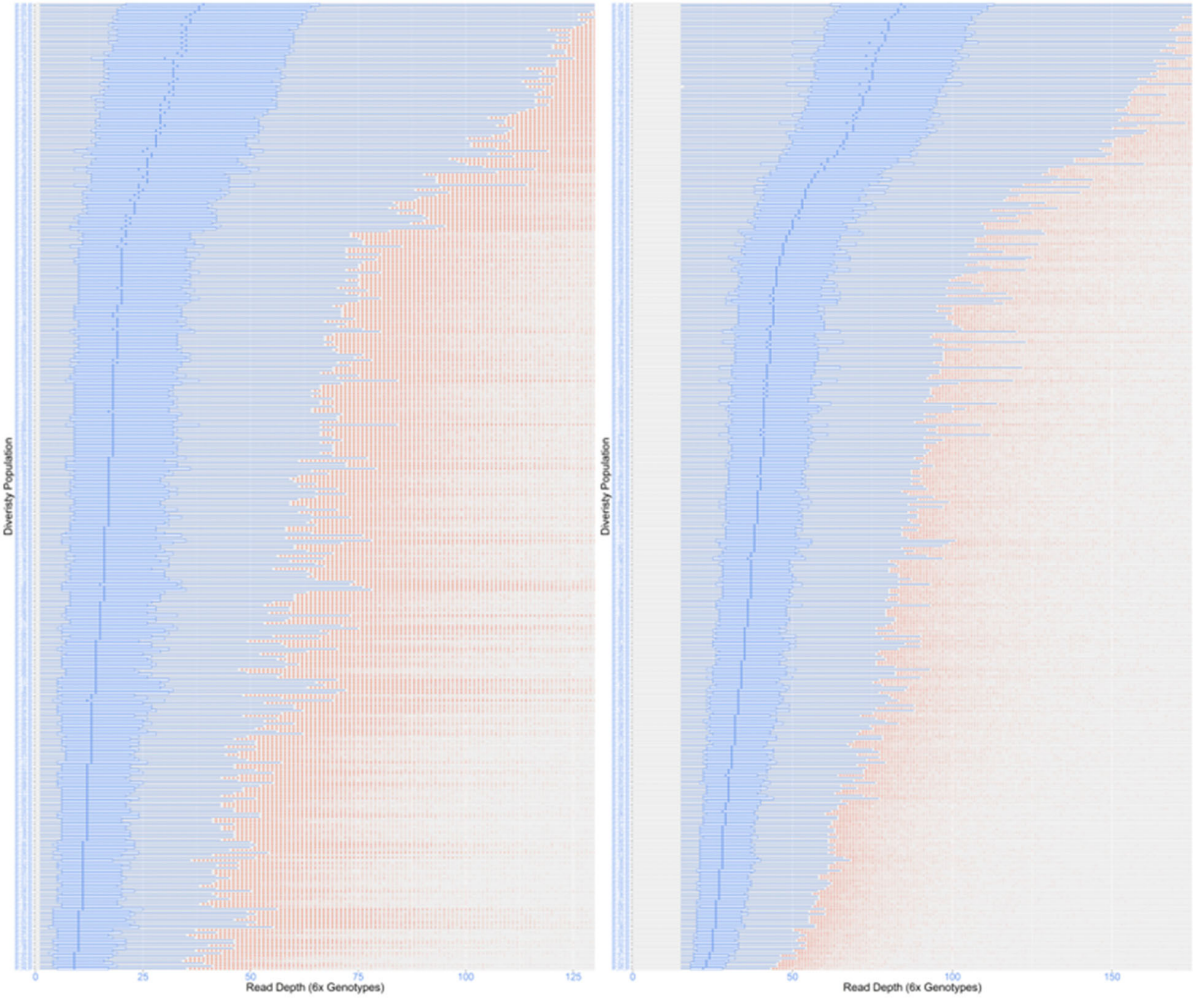
Assessing call coverage quality across clones and loci on raw data. The figure refers to read depth of all reads mapping to all six homeologs

The accuracy of the genotype calls has a fundamental impact on the biological interpretation. We mapped sweetpotato sample reads to the genome assemblies [20] of the putative diploid ancestral progenitors (*I. trifida* and *I. triloba*) in order to determine sequences with 2x, 4x, and 6x doses that correspond to diploid, tetraploid, and hexaploid genotypes in the hexaploid sweetpotato genome. Based on the distribution of read depth for each individual in the population (Fig. 3), the median read depth are about 20x and 40x for before and after applying a 15x read depth threshold, respectively.

We examined genotype quality of variants with dosage and diploidization genotypes using the GBSapp bioinformatic pipeline [20]. At about 85% confidence level and 45x read depth threshold, only 5839 SNPs (without maf filtering) could be called with dosage information, of which 5.54% were multi-dose (duplex and triplex). Genotype calling on a larger set of SNPs resulted in low confidence dosage calls (i.e. about 41,060 SNPs based on a 65% confidence level, 15x read depth threshold and without maf filtering). Considering the limited number of higher confidence dosage-based SNPs, which is suboptimal for genome-wide association analysis, the larger set of dosage-based SNPs with low confidence were diploidized by only scoring genotypes as heterozygous or homozygous. To ensure accurate diploidized calls, the read depth threshold was set to 15x. This resulted in a total of 33,068 SNPs at a minimum minor allele frequency (maf) of 0.05 and no more than 20% missing data.

### Linkage disequilibrium in polyploid sweetpotato

**Fig. 4:**
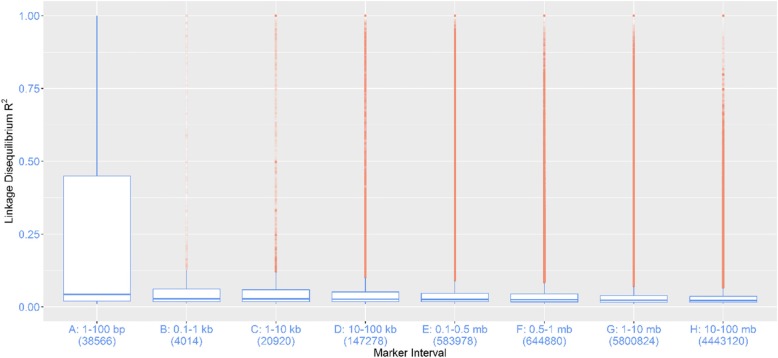
Pattern and extent of genome-wide linkage disequilibrium (LD): boxplots showing the distribution of LD within a range of distances

The pattern and extent of genome-wide linkage disequilibrium (LD), which is the non-random association of alleles at different loci, is presented in a boxplot that shows the distribution of LD within a range of distances between marker pairs (Fig. 4). All pairwise LD (r^2^) values were calculated using the diploidized genotypic data. Overall, based on the *I. trifida* genome assembly size of 526.4 Mb [20] and a total OF 33,068 SNPs (16 Kb marker interval), the marker resolution in this study revealed LD decayed below this distance of 16 Kb and probably at distance less than 1 Kb (Fig. 4). This rapid decay is expected for outcrossing species, especially in polyploids where recombination is elevated post-polyploidization [23]. According to Vos et al. [24] rapid LD decay implies that the resolution for fine mapping causal genes is high, hence, the need to use a high marker density in the form of diploidized SNPs for the genome-wide association analyses.

### Genome-wide SNP data

Out of 33,068 SNPs analyzed in this GWAS, based on Bonferroni correction-based threshold, 34 and seven SNPs were significantly associated with CSRFAB (150 DAP) and DCSRFAB (90 DAP), respectively. The number of significant SNPs associated with the other CSRFAB-related traits (i.e. 90 DAP, 120 DAP, 150 DAP, 180 DAP, slope, and AUGPC) are shown in Table 1. The number of significant SNPs associated with the harvest times (HTs) ranged between 0 (120 DAP) and 34 SNPs (150 DAP). A high marker effect was observed for AUGPC (21.45), followed by 150 DAP (1.23). SNPs associated with 150 DAP and AUGPC explained more phenotypic variation (4.3 to 79% and 12.6 to 24.6%, respectively) compared to other time harvests, while the least phenotypic variation explained was recorded at 120 DAP and 180 DAP (4.4 to 5.3% and 5.3 to 9%, respectively).

**Table 1:** Summary of the SNPs significantly associated with continuous storage root formation and bulking

Trait	Chr	SNPs	p-value Range	maf Range	R^2^ range	Effect range
90 DAP	6	7	2.25E^− 14^ to 1.01 E^−6^	0.03 to 0.49	8.2 to 19.0	− 0.23 to 0.19
150 DAP	14	34	3.75E^−47^ to 9.9 E^−5^	0.02 to 0.49	4.3 to 79	−0.90 to 1.23
180 DAP	1	1	1.12 E^−7^ to 7.2 E^−5^	0.065 to 0.49	5.3 to 9	0.03 to 0.20
Slope	8	9	2.5 E^−10^ to 9.9 E^−5^	0.028 to 0.49	7.6 to 15	−0.52 to 0.55
AUGCP	6	6	1.7E^−14^ to 3.8 E^−5^	0.03 to 0.49	12.6 to 24.6	−15.20 to 21.4
Total	14	57	3.75E-47 to 3.8 E-5	0.02 to 0.49	4.3 to 79	−15.20 to 21.45

*Chr* Chromosome, *SNPs* Number of single nucleotide polymorphisms, *PV* p-value, *maf* minor allele frequency, *R*^*2*^ percentage of phenotypic variation explained

Among the 57 SNPs associated with CSRFAB and DCSRFAB at different storage root development stages, some SNPs were shared between 150 DAP, AUGPC and 180 DAP, while other SNPs were simultaneously detected between 90 DAP, 120 DAP and slope. The largest number of associated SNP markers was detected on chromosome 1 and the largest number of significant associations was recorded for 150 DAP followed by the slope (Table 1). Most of significant SNPs associated with 90 DAP and slope were co-located while significant SNPs associated with 150 DAP and AUGPC were co-located (same or close position) on chromosomes.

Thirty-four SNPs (around 60% of the significant SNPs) were associated with CSRFAB scores collected at 150 DAP and were concentrated on chromosome 1, 6 and 8. All the minor allele frequencies (MAFs) of the significant SNPs at Bonferroni correction rate of 5% were above 10%, equivalent to recommended strict threshold for MAFs. All the significant SNPs passing Bonferroni threshold of 5% scored low adjusted *P*-values (FDR adj P-values) ranging from 8.11E-43 to 1.79E-03, implying that the type I error with false positive SNPs was minimized. The top four most confident SNPs discoveries were Chr04_6057850, Chr12_903336, Chr01_30468732, and Chr09_6258404. Most significant SNPs that passed the stringent significance threshold of 5% Bonferroni were also associated with CSRFAB variations at 150 DAP and R^2^ were greater than 9% and the majority explained more than 15% of the variation. In the previous study, 150 DAP and 90 DAP were proposed to predict CSRFAB and DCSRFAB traits and further investigation in this study will be only focused on these two times [3].

### Significantly associated SNPs

**Fig. 5:**
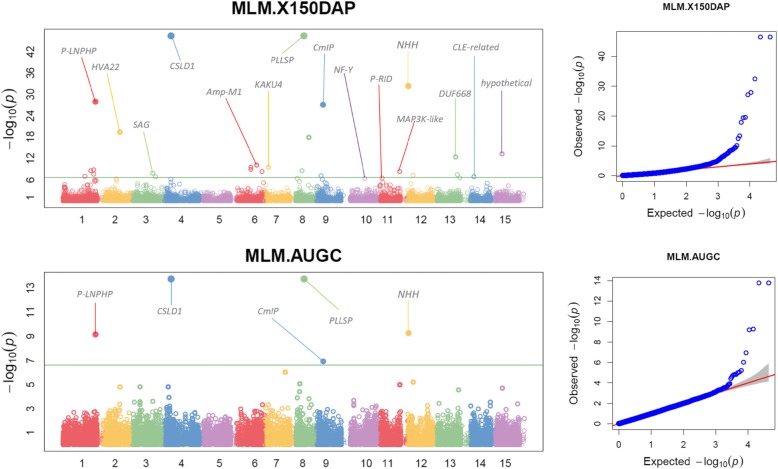
Manhattan plots at 150 days after planting and area under growth progress curve (left), and corresponding quantile-quantile plots (right). Note: CSLD1 = Cellulose synthase-like D1; NHH = Nudix hydrolase homolog; P-LNPHP = P-loop containing nucleoside triphosphate hydrolase superfamily protein; CmIP = Cam interacting protein; Amp-M1 = Aminopeptidase M1; P-RID = Putative recombination initiation defect; PLLSP = Pectin lyase-like superfamily protein; HVA22 = HVA22 homologue A; SAG = Senescence-associated gene; NF-Y = Nuclear factor Y, subunit B4; MAP3K-like = Mitogen-activated protein kinase kinase kinase-like; CLE-related = CLAVATA3/ESR-like

**Fig. 6:**
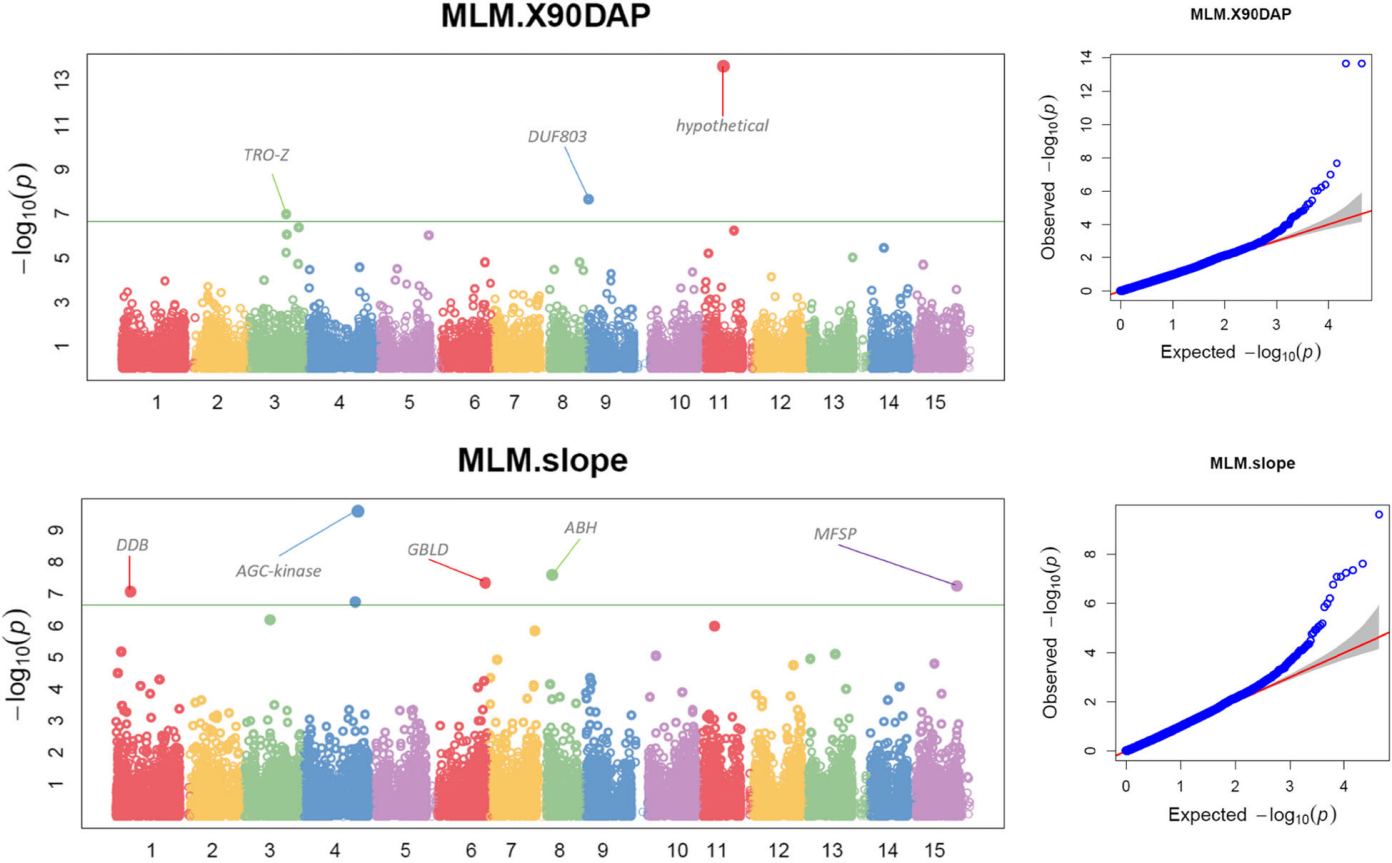
Manhattan plots at 90 days after planting and slope (left), and corresponding quantile-quantile plots (right). Note: TRo-z = Thioredoxin Z; AGC-kinase = cAMP-dependent, cGMP-dependent and protein kinase C kinase family protein; GBLD = Golgi-body localization protein domain; ABH = Alpha/beta hydrolase; MFSP = Major facilitator superfamily protein

The GWAS results were visually examined using Manhattan plots and quantile-quantile (QQ) plots [25]. The Manhattan plots (Figs. 5 and 6) show on the y-axis the negative log-base-10 of the *P* value for each of the SNPs in the genome (along the x-axis), when tested for differences in frequency between trait and markers. The line shows the threshold for genome-wide significance (*P*-value < 5 × 10^− 7^). Each dot is a SNP laid out across the sweetpotato chromosomes from left to right, and the heights correspond to the strength of the association to traits under study (150 DAP and AUGPC see Fig. 2; 90 DAP and slope see Fig. 6). Large peaks in the Manhattan plot were observed at 150 DAP and 90 DAP. The lines show that most of the significant SNPs at 150 DAP and AUGCP are on the same chromosomes, although the strength of the association signal is different. The significant SNPs at 90 DAP and slope are consistently on the same chromosomes with consistent reduced -log_10_ of P-value for slope. Our results show a major locus on chromosome 1 (GAPIT output https://data.cipotato.org/dataset.xhtml?persistentId=doi%3A10.21223%2FKM16BH). Chromosomes 1, 3, 4, 6, 8 and 12 had a high number of the associations over the four harvest times forming clusters where multiple traits were associated due to probably a set of linked genes. These regions include: chromosome 1 (six traits), chromosome 3 (4 traits), chromosome 4 (4 traits), and chromosome 12 (four traits). On a multiple plot, we placed vertical lines at specific positions along the x-axis to identify consistently associated QTNs (Figs. 5 and 6). The Q-Q plot was used to evaluate the false positive rate (spurious association due to confounding factors) and to compute adjusted *p*-values. The observed –log_10_ *P*-values which indicate the significance level of association statistics are displayed in Figs. 5 and 6 and are ranked from the smallest to the largest on the y-axis. The corresponding SNP markers are plotted against the distribution that would be expected to have no association on the x-axis under the null hypothesis. It is expected that the deviations from the identity line observed values contain potential true associations. The strength of the association signal is displayed in two ways. One indicator of strength is the height on the vertical axis for –log_10_ P-values; the greater the height, the stronger the association. The height on the vertical axis for –log_10_ P-values was high for 150 DAP followed by 90 DAP and AUGPC.

### Candidates co-localize with associated SNPs

GWAS are useful for identifying genomic locations related to a trait of interest, however, the information provided does not capture the function of genes under the region of interest. It is therefore of great importance that putative causal gene functions be determined and investigated through different functional genomic approaches. It is well recognized that few of associated markers involve genes previously reported to be related to the trait of interest, and some are found in genomic locations harboring no known genes and sometimes regulatory elements [25]. Therefore, the genomic locations of our significant SNPs were investigated to identify which protein-coding genes the SNPs were located in or adjacent to, using the online database of *I. trifida* reference genome (http://sweetpotato.plantbiology.msu.edu/). Candidate genes that are co-located with the top significant SNPs are listed in Table 2.

**Table 2:** Significant marker-trait associations, candidate genes and corresponding putative functions for continuous storage formation and bulking (CSRFAB) and discontinuous storage root formation and bulking (DCSRFAB)

Variants^1^	*P*-value	R^2^	Allele effect	maf^3^	Orthologs	Putative function
Continuous Storage Formation and Bulking (CSRFAB; 150 DAP)						
Chr04_6057850	3.7E-47	0.8	0.14	0.24	Cellulose synthase-like D1	Root hair development.
Chr12_903336	4.8E-33	0.5	0.35	0.39	Nudix hydrolase	Excessive cell stimulation and stress response.
Chr01_30468732	1.4E-28	0.41	−0.27	0.37	P-loop containing nucleoside triphosphate hydrolases	Regulates senescence, cell death and stress.
Chr09_6258404	8.8E-28	0.4	−0.19	0.15	Calmodulin Interacting protein	Regulates plant shoot branching.
Chr02_16003392	4.8E-20	0.27	0.04	0.49	HVA22 homologue A	Stress-induced programmed cell death.
Chr08_14465821	8.6 E-06	0.12	0.16	0.03	Pectin lyase-like	Cell wall remodeling, growth and senescence.
Chr06_13670137	3.2E-10	0.12	0.13	0.49	Aminopeptidase M1	Plant growth, leaf longevity and stress response.
Chr07_3326491	3.5E-10	0.12	0.36	0.04	KAKU4	Modulates nuclear morphology
Chr11_18261627	5.6E-09	0.1	0.10	0.49	Putative recombination initiation defect	Meiotic recombination
Chr03_18988293	1.5E-08	0.09	−0.09	0.50	Senescence-associated gene	Programmed cell death
Chr14_3298542	1.3E-07	0.08	0.27	0.49	CLAVATA3/ESR-RELATED	Meristem maintenance
Chr11_1919638	3.3E-07	0.08	0.16	0.49	MAP 3 K-like	Plant growth, development and stress response.
Chr10_13852642	3.8E-07	0.08	0.12	0.50	nuclear factor Y, subunit B4	Plant growth, development and stress response.
Discontinuous Storage Formation and Bulking (DCSRFAB; 90 DAP)						
Chr03_18599676	1.10E-07	0.1	−0.18	0.14	Thioredoxin z	Reduction of oxidative stress.
Chr09_299562	2.30E-08	0.1	−0.02	0.11	DUF803	Unknown protein
Chr11_14588348	6.20E-07	0.09	−0.03	0.17	hypothetical	Reduction oxidative stress.
Chr03_18916775	9.30E-07	0.08	0.04	0.49	AGC-Kinase	auxin-induced lateral root formation.
Chr06_2448368	4.6 E-08	0.08	−0.24	0.27	GBLD	Stress, signal transduction.

^1^chromosome and variant position, i.e. SNP and indels (*)

^2^proportion of phenotypic variation explained

^3^minor allele frequency

Candidate genes that might underlie variation in CSRFAB were identified based on proximity of the associated SNPs with predicted genes (Table 2). The strongest association detected for CSRFAB was observed at 150 DAP (PV = 3.7E-47), on chromosome 4 and was in strong LD with a cellulose synthase-like D1 gene involved in root hair development [26]. Another highly significant SNP (PV = 4.8E-33) was located on chr12 and was in strong LD with a coding region (exon) of a NHH gene that belongs to the family of nudix hydrolase homolog having Nudix box-containing proteins previously reported in plant defense responses against biotic and abiotic stresses [27].

The associated Chr01_30468732 SNP is co-localized with P-LNPHP gene encoding a P-loop containing nucleoside triphosphate hydrolases super family protein involved in regulation of growth and hormonal signaling in plants. The gene HVA22 was found to be associated with Chr02_16003392 locus encoding basic helix-loop-helix bHLH-DNA-binding superfamily protein and was previously reported to regulate leaf senescence, cell death and abscisic acid (ABA) biosynthesis [28]. Annotated SAG gene was found to be associated with Chr03_18988293 locus and was encoding Transducin/WD40repeat-likesuperfamily proteins known as key regulators of plant-specific events, biologically playing important roles in development and also during stress signaling [29]. The gene CSLD1 was co-located with SNP Chr04_2472869 and this gene encodes for cellulose synthase-like D1 reported to have pleiotropic effects on multiple agronomic traits that alter plant organ size by changing the process of cell division [30] and is required for root hair morphogenesis in Arabidopsis [26]. Other associated SNPs with CSRFAB including Chr06_13670137, Chr07_19060345, Chr08_14465821, Chr10_5821301, Chr11_18261627, Chr12_903336 and Chr12_5341809 were co-located or adjacent to AMP-M1, KAKUA4, PLLSP, CMmIP, NF-Y, P-RID, NHH, MARP3K-like, DUF668, CLE-related and hypothetical, respectively. These genes encode respectively Aminopeptidase M1 that regulate plant growth, leaf longevity and stress responses, ProteinKAKU4 playing a role in modulating nuclear shape and size usually impacting adaptation to stress, DHHC-typezincfinger family protein regulating senescence, cell death and shoot branching in Arabidopsis [31], HVA22 homologue A having important roles in plant stress-induced programmed cell death and leaf senescence [32], terpene synthetase, a precursor of gibberellins with essential roles for plant growth and development, putative recombination initiation defect protein, playing important roles throughout plant growth and development [33], nudix hydrolase homolog, involved in resistance to biotic and abiotic stresses [34] and conserved hypothetical protein found among the top 70 up-regulated contigs in initiating storage roots compared to fibrous roots in sweetpotato [35]. All these genes were associated with 150 DAP and AUGPC and were different from those observed at 90 DAP and the slope.

### Specific genes detected for DCSRFAB during sweetpotato storage root development

Candidate gene TRO-Z was associated with Chr03_18599676 SNP marker and this gene encodes thioredoxin z previously reported as a facilitator of plants to cope with fluctuating environments by integrating energy transduction, metabolism, gene expression, growth and development [36]. The gene, AGC-kinase, was adjacent to Chr03_18916775 locus and encodes Autoinhibited Ca(2+)-ATPase, playing a role in sucrose signaling during early seedling development by integrating developmental signals with carbon source availability [37]. The gene,, GBLD, was associated to Chr06_22463100 SNP and encods transglutaminase-like superfamily domain containing proteins known to have a regulation role in defense and stress response systems across the tree of life [38] and ABH gene was associated with Chr07_22973475 locus and encodes alfa/beta hydrolase serving as the core structure for phytohormone and ligand receptors in the gibberellin, strigolactone, and karrikin signaling pathways in plants and has evolved complex and specialized chemical adaptations for survival responses to widely varying biotic and abiotic ecologies [39]. The gene, DUF803, hypothetical and MFSP were involved in gibberellic acid (GA) and abscisic acid (ABA) signaling in the regulation of growth [40], the MFSP are transporters of small solutes in response to chemiosmotic ion gradients allowing the uptake of essential nutrients and ions in plants [41]. Most of these genes are summarized in Table 2 below.

### Shared genes detected during sweetpotato storage root development

**Fig. 7:**
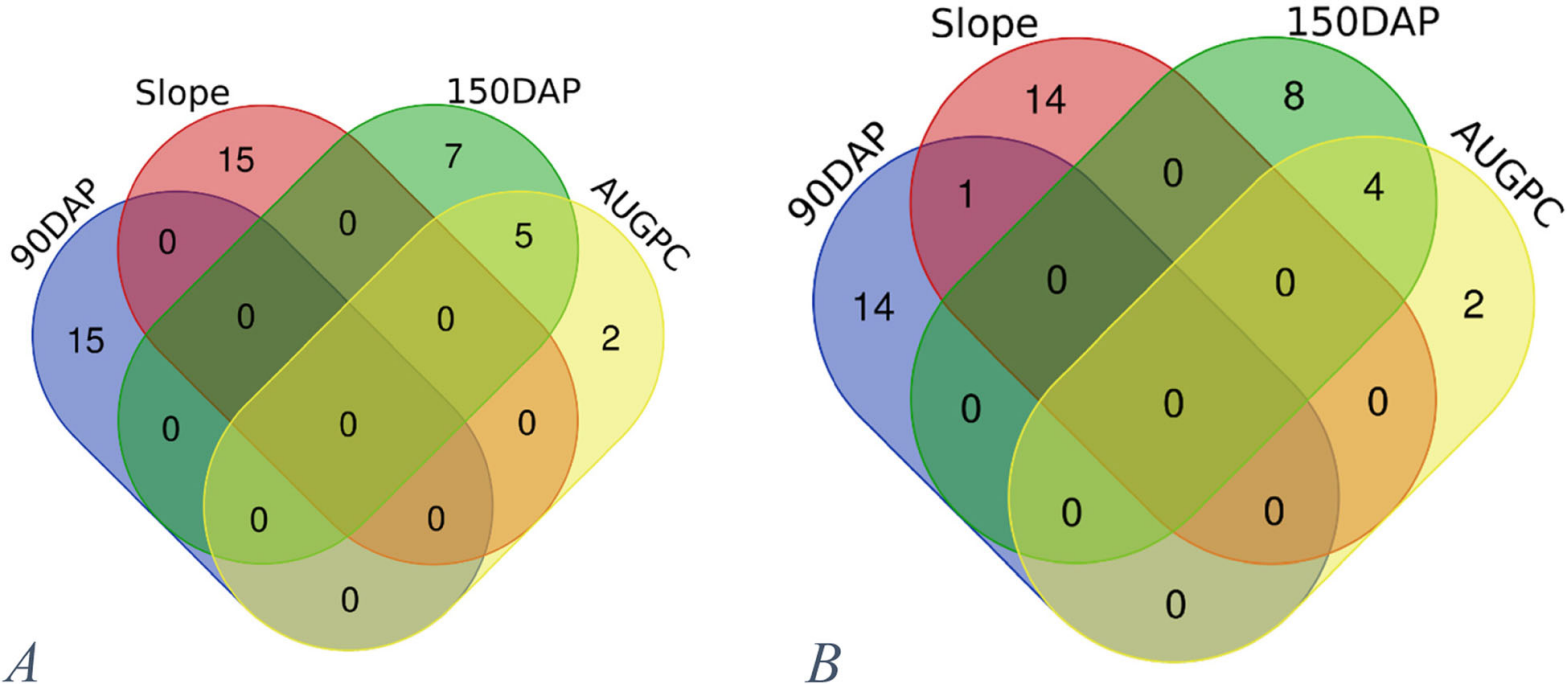
Venn diagrams showing common loci identified in this study. **a**. Common locus among four traits. **b**. Common genes among four traits: DAP = days after planting, AUGPC = area under growth progress curve

The Venn diagrams (Fig. 7) show how traits share SNPs (Fig. 7a) and associated genes (Fig. 7b). The venn diagram (Fig. 7a) shows that QTNs identified for 90 DAP and 150 DAP are completely different indicating a different gene regulation system for the two phases. The slope and 90 DAP have common SNPs. However, most of the common SNPs had reduced *P*-values for the slope, indicating that slope is not a good predictor of responses at 90 DAP. Five genome wide SNPs hit common chromosome region at 150 DAP and AUGPC (Fig. 7a), whereas seven and two QTNs were unique to 150 DAP and AUGPC. Four nearest genes were commonly identified for 150 DAP and AUGPC and none common nearest genes at 90 DAP and slope, confirming the findings in 7. A.

## Discussion

### Correlation and variability analysis

In this study, we showed that CSRFAB, SRN, SRY and HI were highly correlated, suggesting that their genetic basis is controlled, in part, pleiotropically (i.e. some alleles affect two or all the four traits involved in CSRFAB). This implies that we can expect positive changes in the SRN and SRY for varieties whose CSRFAB expression is increased. In breeding, this means that when we select for CSRFAB we can get a correlated response in the SRN and SRY. Further detailed analyses on the CSRFAB trait responses over the four harvest times and their respective predictor (AUGPC and slope) revealed that AUGPC and 150 DAP were highly and positively correlated (R^2^ = 0.91). This indicates that AUGPC and 150 DAP tightly affect the same phenotype. In a previous study [3], 150 DAP was suggested as the scoring time for CSRFAB in sweetpotato due to its potential to account for regrowth and growth cessation in two different environments in Uganda. The genetic correlation between a phenotypic trait implies that indirect selection is possible and is an attractive alternative to direct selection and it should not be overlooked in breeding strategies.

We showed high variability for CSRFAB at the different harvest times and this variability has a temporal growth pattern which was more pronounced at two harvest times (90 DAP and 150 DAP). In a previous study [3] these harvest times were critical for characterizing the type of growth. For the 150 DAP genotypes, logarithmic growth increased the speed of growth and peaked at 90 to 120 DAP and then decreased their growth rates. In the 90 DAP genotypes, changes in the later phase or growth were more variable in their growth patterns. These results indicate that selection aimed at improving CSRFAB would increase storage root yield in sweetpotato. Previous studies by Egbe et al. (2012) [42], Afuape et al. (2011) [43], and Thiyagu et al. (2000) [44] concur with our findings and attributed yield increment to increase in both number of storage roots and weight of individual storage roots. The correlation between CSRFAB and 150 DAP indicates that we can select CSRFAB genotypes using score responses at 150 DAP. These correlation coefficients are important in breeding and provide a means to associate characters in breeding and selection for a given character.

### Association analysis

We used genome-wide association (GWA) analysis to identify SNPs associated with CSRFAB and DCSRFAB in sweetpotato. GWA analysis provides much more precise physical positioning than the method of QTL mapping in biparental mapping populations that has been previously used for complex growth traits in plants. We observed particularly high *P*-values (ie.3.75E-47, 4.8E-33, 1.39E-28, 8.83E-28) suggesting the presence of some major to moderate QTL particularly underlying CSRFAB (the wild type or predominant habit of sweetpotato), while DCSRFAB (the habit bred for in cultivated sweetpotato) seems to be controlled by minor alleles. The rapid decay of LD in the diversity panel breaks the genome in small LD blocks and provides us with the ability to fine map QTL, oftentimes to the gene level [45]. In this set of sweetpotato clones, chromosomes 1, 3, 4, 6 and 12, that harbour CSRFAB loci, were among the five chromosomes exhibiting the most extensive LD [46]. We detected 34 SNPs mapped on 150 DAP and clusters of SNPs consistently mapping similar SNPs to 180 DAP and AUGPC. Most of the SNPs associated with 150 DAP, 180 DAP and AUGPC were in high LD, however, the P-values recorded on the SNPs associated with 180 DAP and AUGPC were reduced making some of them not significant at 5% Bonferroni threshold. Thus, it is possible that SNPs associated with 150 DAP, 180 DAP and AUGPC are associated with the same underlying causal variation. Likewise, SNPs associated with DCSRFAB (90 DAP, 120 DAP and slope) were also in high LD. However, no overlap was observed between SNPs of one cluster to the other, suggesting that SNPs identified for CSRFAB and DCSRFAB are associated with distinct causal polymorphisms.

### Candidate genes

We used the publicly available *I. trifida* genome sequence to identify candidate genes encompassing or adjacent to these SNPs. Several of the candidate genes that we identified play a role in the plant growth pathways. Candidate genes can also be identified based on their positions on quantitative trait locus (QTL) maps or patterns of gene expression [47]. Identified genes were involved in the expression of a phenotype affected by many genes with small effects. This nature of gene pattern is reported in adaptive complex traits like CSRFAB [48]. Suzuki (2017) [48] argued that traits are partly decomposable into an assembly of subcomponents. Intermediate versions of complex traits exist in extant species like in sweetpotato where Lee et al., 2012 [49] classified sweetpotato cultivars into wild and modern types based on the expression of storage root bulking. Interestingly, such intermediate phenotypes are formed from different combinations of homologous subcomponents (alleles) with some modifications or the addition of new components. It is known that major genes almost always have multiple effects (pleiotropism), which can simultaneously convey separate advantageous traits and disadvantageous traits upon the same organism [50]. In this instance, the state of the sweetpotato as hexaploid crop provided selection, with a net effect favoring the genes for CSRFAB to survive in nature. Most areas of growth follow two different types of growth: a logarithmic and an exponential growth curve. The logarithmic growth curves which characterize DCSRFAB [3] increase quickly in the beginning, but the gains decrease and become more slow as time goes on, while the exponential growth curves (CSRFAB) increase slowly in the beginning, but the gains increase rapidly and become larger as time goes on. This basic principle in plants is complex and occurs in CSRFAB in sweetpotato. Identified genes involve growth hormones such as auxins, gibberellins and ethylene signaling. For instance, Numerous observations suggest a tight correlation between auxin, ethylene and lateral root formation. These hormones have been previously reported to be involved in regulation of stay-green processes in plants [51] by maintaining greenness of leaf or by initiation and progression of leaf senescence. At low levels, ethylene promotes auxin biosynthesis and/or response, and promotes lateral root initiation in young root portions. Upon an increase in the level of ethylene, ethylene interacts with auxin in the tip of the primary root and suppresses root growth. This inhibits lateral root initiation in root regions with inhibited growth. Simultaneously, ethylene promotes the emergence of existing lateral root primordia. Ethylene is produced in all parts of the stressed plant and auxin is formed in apical meristems and is transported through phloem to roots [52]. The cluster of SNPs associated with DCSRFAB were mostly associated with ethylene biosynthesis (i.e. P-loop containing nucleoside, Nudix hydrolase, DHHC-type-zinc finger, HVA 22 homolog A, Ca^2+^ dependent kinase) and stress signaling pathways whereas the cluster of genes associated with CSRFAB involved mostly growth hormone signaling such as auxin, ABA, gibberellins (i.e. Cellulose synthase-like D1, Aminopeptidase M1, calmodulin interacting protein, autoinhibited Ca^2+^ − ATPase). The former modulate growth that is declining due to senescence, aging, and drought stress. ABA is reported to be involved in the repression of germination, drought response, and promotion of leaf senescence [51].

This situation makes continuous storage root growth regulation a highly complicated process and it seems to be controlled at many different levels by complex actions of gene networks in both time and space. For instance, SNPs as well as genes involved in storage root formation and bulking at 90 DAP were completely different from genes found at 150 DAP implying that the regulation mechanism of root formation and bulking is time dependent.

A key contribution of this study is the multipurpose characteristic of the discovered SNPs that can be validated for early maturing and continuous storage root formation for commercial storage root production, piecemeal harvesting and biomass production for animal feed. At the top of significant and upregulated genes, P-loop containing nucleoside triphosphate hydrolases super family protein and DHHC-type zinc finger family protein were associated with CSRFAB in this study and these genes generally have a meristem-specific mRNA expression pattern and KNOX proteins playing central roles in shoot development by maintaining the apical meristem activity [53]. Cellulose synthase-like D1 might be involved in storage root formation. This is because it has been reported to be implicated in organ size and root growth regulation [30]. Storage root bulking is a complex process that consists of cell division and expansion. Having these genes upregulated at 150 DAP is an indication of continuous biological activity in the plant having an implication of differentiation of organs in the plant. Furthermore, our study has identified genes associated with CSRFAB that have a prominent role in ABA biosynthesis and other hormones signaling such as ethylene, cytokinin, gibberellin and auxin in sweetpotato [54, 55, 19]. Ravi et al., [55] reviewed the molecular physiology of storage root formation and development in sweetpotato and produced several reports that suggest a relationship between storage root formation (initiation) with cytokinins and several cytokinins were involved in sweetpotato storage root formation by developing and activating the primary cambium [56]. Our results are consistent with all these reports, which indicates that the detected differentially expressed genes during storage initiation and development would be of great value in uncovering molecular mechanisms relating to continuous storage root formation, bulking and further development.

## Conclusion

We genotyped 358 sweetpotato genotypes and run the first GWAS in sweetpotato for CSRFAB. The study identified 40 unique SNPs significantly associated with CSRFAB traits and 12 unique SNPs significantly associated with DCSRFAB. Novel genes including 12 promising genes for CSRFAB and seven genes for DCSRFAB were identified in this study. These genes can be used for sweetpotato genetic improvement of CSRFAB using maker assisted breeding approaches after marker validation. To validate these associations and candidate genes, additional studies will be required (e.g. transcript/transcriptome analysis, increasing maker density for fine mapping, and mapping in other mapping populations). The discovery of the candidate genes has increased our understanding of the molecular causal mechanisms of CSRFAB in sweetpotato and may contribute to the basic knowledge in breeding for CSRFAB in sweetpotato. The validation of genotypes with excellent haplotypes will provide valuable breeding materials to improve sweetpotato for CSRFAB through marker-assisted selection in future breeding efforts.

## Methods

### Plant materials, field trials and phenotypic evaluation

Parental genotypes were obtained from the National Crops Resources Research institute (NaCRRI) in Uganda. The study involved a collection of 358 sweetpotato genotypes including; a set of 130 genetically diverse genotypes from two contrasting genepools previously characterized with 31 simple sequence repeat (SSR) markers [57] and a set of 228 genotypes that were derived from crosses using 20 parents, selected from the above set of 130 genotypes. The 20 selected parents were comprised 10 CSRFAB and 10 DSCRFAB genotypes, while the 228 derived F1 genotypes segregated for the two traits. The 228 progenies were generated by hand pollination using North Carolina II mating design [58]. Trials were conducted at NaCRRI (Namulonge, Uganda) and the National Semi-arid Resources Research Institute (NaSARRI, Serere, Uganda) during the long rainy season, starting from March (2017), and the short rainy season starting from September (2017). The coordinates of Namulonge site are at latitude 0.5250, longitude 32.6150, at an altitude of 1150 m.a.s.l., while the Serere site is at latitude 1.4970, longitude 33.3935 and at an altitude of 1140 m.a.s.l.

The experimental design was based on a randomized complete block design, with a two-row experimental plot containing 20 plants. A total of 2 replications were sampled even though 4 replicates were planted. Measurements were conducted at one-month intervals starting from 3 months after planting (MAP).

### Phenotypic data collection

To identify the patterns of storage root formation and bulking, storage roots for each plant genotype were destructively sampled at 3, 4, 5, and 6 MAP. At each sampling point, data were collected from four plant stands using above- and below-ground parts. The following sweetpotato storage root traits were measured for each of the four harvest times to allow correlation analysis of CSRFAB and yield component traits and these included: (i) number of harvested plants (NPH), allowing calculation of average values, (ii) total storage root number (SRN), (iii) total root weight (TRW), and (v) vine weight (VW). CSRFAB was estimated using a previously developed scale of 1 to 9, where 1 = no visible storage root (SR) initiation and no visible bulking; 2 = No visible SR initiation but bulking is detectable; 3 = No visible SR initiation; 4 = Distinct SR initiation, with 2 bulking roots; 5 = Distinct SR initiation and 3 bulking roots; 6 = Distinct SR initiation and 4 bulking roots; 7 = Distinct SR initiation and 5 bulking roots; 8 = Distinct SR initiation and 6 bulking roots; 9 = Distinct SR initiation and 7 bulking roots [3].

### Predicting growth, growth predictors identification and estimation

Most growth curves in living organisms follow a logarithmic or exponential growth trend. The logarithmic growth curves increase quickly in the beginning, but the gains decrease and become slower as time goes by, while the exponential growth curves increase slowly in the beginning, but the gains increase rapidly and become larger as time goes [59]. The increase in growth rate is described by its slope and a measure of the total growth is described by the area under the growth progress curve [60]. These two variables were explored to investigate the possible indirect selection of the traits associated with CSRFAB. CSRFAB has previously showed high correlations with yield and yield components such as storage root number and storage root diameter which indicate that genetic control is tightly linked with those traits and indirect selection of high yielding varieties can be based on the CSRFAB trait [3]. Thus, CSRFAB scores throughout the study were used to estimate such phenotype. Overtime, CSRFAB scores were therefore converted to AUGPC and the instantaneous rate of change or slope of a function underlying the four data points. These two parameters along with the four harvest time responses were used to characterize CSRFAB change over the four harvest times. The formulas used for AUGPC and slope were as follows:

$AUGPC=\sum \limits_{i=1}^{n-1}\frac{Y_i+{Y}_{i+1}}{2}\left({T}_{i+1}-{T}_i\right)$ where Yi is the given score at harvesting time i and Ti is the harvesting time i [61].

Slope: m = f ‘(x) = $\underset{h\to 0}{\lim}\frac{\int \left(x+h\right)-f(x)}{h}$ where f ‘(x) is called the derivative of the function f with respect to x; x is the time of harvest and h is a constant [60].

### Statistical analysis of phenotypic data

The analysis of variance was performed for CSRFAB scores, slope and AUGCP predictors that take into account all the time harvests at once. R package lme4 [62] was used for statistical analysis. The model for the phenotypic data analysis was *Y*_*ijk*_ = *μ* + *G*_*i*_ + *T*_*j*_ + *E*_*k*_ + *GT*_*ij*_ + *GE*_*ik*_ + *GTE*_*ijk*_ + *R*_*lk*_ + *e*_*ijkl*_; where μ is the total mean, Gi is the effect of the ith genotype, T_j_ is the effect of the j^th^ harvesting time, GT_ij_ is the effect of the interaction between the i^th^ genotype and the j^th^ harvesting time, GTE_ijk_ is the effect of the interaction between the i^th^ genotype and the j^th^ harvesting time in the k^th^ environment, E_k_ is the effect of the k^th^ environment, GE_ik_ is the interaction effect between the i^th^ genotype and the k^th^ environment, R_ik_/ is the effect of the L^th^ block within the k^th^ environment, and e_ijk_ is a random error .

Correlation analyses between CSRFAB traits were performed using the cor function and Pearson method in R software.

Boxplots [63] were used to display a five-number data summary to better describe the variability within the data. The aesthetics with aes() function together with the geom_boxplot() layer both in ggplot2 (performed in Rstudio) provided a visualization of the data variability and dispersion.

### DNA extraction

A total of 5 mg of young leaf tissue was sampled for each genotype using a polythene sampling bag and then kept cool under ice. Extraction buffer was prepared (200 mM Tris-HCl, 50 mM EDTA, 2 M NaCl, 2% CTAB, and 3% β-mercapto ethanol) and placed in a water bath at 65 °C. Using a FastPrep-24™ 5G tissue homogenizer, leaf tissue samples in 2 ml tubes were ground for 5 min in liquid nitrogen using sterile 4 mm stainless steel ball bearings. To obtain high molecular weight DNA, 1 ml of prewarmed (65 °C) CTAB buffer (200 mM Tris-CL, 50 mM EDTA, 2 M NaCl, 2% CTAB and 3% β-mercapto-ethanol) was added to the ground samples and vortexed at 3000 rpm for 30 s. Tubes were heated in a water bath at 65 °C for 30 min and mixed gently at 10 min interval during incubation. Samples were cooled on ice, 500 μl chloroform:isoamyl alcohol (24:1) was added, and mixed by inverting the tubes 20 to 30 times. Samples were then centrifuged at 15,000 round per minute (rpm) for 15 min and the top layer recovered into a new tube. This step (chloroform:isoamyl alcohol) was repeated to ensure purity of extracted DNA. DNA was precipitated with 1/5 volume of 5 M NaOAC and 2.5 volume of cold absolute ethanol (stored at − 20 °C). The samples were gently inverted to mix and incubated at − 20 °C for 60 min. Samples were centrifuged and DNA pellet recovered by decanting the supernatant. The DNA pellet was washed twice with 500 μl of cold (− 20 °C) 70% (v/v) ethanol and air dried. The DNA pellet was resuspended in 100 μl low-EDAT TE buffer (1 mM Tris-Cl, 0.1 m M EDTA) containing 400 μg RNase-A. The DNA concentration and purity were determined using a NanoDrop spectrophotometer.

### Genotyping, SNP calling and haplotype estimation

DNA samples (40 μl each) were sent to the integrated genotyping services and support (IGSS) at the Biosciences eastern and central Africa - International Livestock Research Institute (BecA-ILRI) Hub for sequencing based on the DArTseq technology. The raw Fastq files were processed within the GBSapp pipeline for pre-processing fastq files, variant and dosage calling, and variant filtering. The pipeline integrates various software, including GATK v3.7 [64], optimized for highly heterozygous and polyploid species [21]. Filtering parameters included read depth filtering for each data point (genotypes with read depth less than the threshold were coded as missing). Also, markers with > 20% missing data, and minor allele frequency < 5% were removed. Out of 46,007 diploidized SNPs, 33,068 informative diploidized SNPs derived from 358 diverse genotypes were considered after the filtering and data quality control process. The two physical reference genomes of sweetpotato’s putative ancestral diploid progenitors, *I. trifida* and *I. triloba* [20] were used for variant calling.

### Linkage disequilibrium

Linkage disequilibrium analysis was performed using GAPIT [65] and implemented in the R-package v3.5.1 using the selected 33,068 SNP markers. Linkage disequilibrium (LD) was estimated as squared allele frequency correlations (R^2^), and only *P*-values < = 0.01 for each pair of loci were considered significant. The LD decays were computed for LD-based genome-wide association analysis.

### Genome-wide association studies

To minimize false positive rates and increase statistical power, the population structure Q and kinship (K) matrix were estimated. A compressed mixed linear model (CMLM) was used, with the kinship or relatedness (K) matrix as a random effect to account for population structure and reduce spurious associations. The analysis was performed using the R package for Genome Association Prediction Tool (GAPIT) version 3 [66]. Variance–covariance kinship matrix (K) was calculated using the VanRaden method [67]. The first three principle components of the dataset were automatically calculated in GAPIT to visualize the genetic diversity across the collection (*N* = 358). The first three principal components of the SNP data were included in the GWAS model. The Bonferroni threshold for *P* values was calculated based on the number of markers (*P* = 1/n, n = total SNP used) according to the method described in Li et al., (2013) [68].

### Identification of candidate genes

Based on the significant trait-associated SNPs, the physical genome assembly of the diploid *I. trifida* (http://sweetpotato.plantbiology.msu.edu/) was used as the reference genome for identifying candidate genes. The putative function candidate genes that co-localized with associated SNPs were annotated based on similarity to known annotated genes in other species, particularly, *Arabidopsis thaliana*. Independent analyses were performed using the Basic Local Alignment Search Tool from the National Center for Biotechnology Information Basic Local Alignment Search Tool (NCBI BLAST) and the conserved domains database (CDD) resources [69] for annotation of the sweetpotato genes. Additional annotation of the candidate genes was confirmed based on review of relevant literature.

## Data Availability

The datasets generated and analysed during the current study are available from the International Potato Centre (CIP) Dataverse: Dataset for Genome-Wide Association Study Identified Candidate Genes Controlling Continuous Storage Root Formation and Bulking in Hexaploid Sweetpotato. https://data.cipotato.org/dataset.xhtml?persistentId=doi%3A10.21223%2FKM16BH).

## Abbreviations

°C: Degree celcius
ABA: Abscisic acid
ANOVA: Analysis of variance
AUGPC: Area under growth progress curve
BecA-ILRI: Biosciences eastern and central Africa - International Livestock Research Institute
BLAST: Basic Local Alignment Search Tool
CDD: Conserved domains database
Chr: Chromosome
CMLM: Compressed mixed linear model
CPK: Creatine phosphokinase
CSRFAB: Continuous storage root formation and bulking
CTAB: Cetyl trimethyl ammonium bromide
DAP: Days after planting
DArTseq: Diversity array technology sequencing
DCSRFAB: Discontinuous storage root formation and bulking
DMC: Dry matter content
DNA: Deoxyribonucleic acid
EDTA: Ethylenediaminetetraacetic acid
GAPIT: Genome Association and Prediction Integrated Tool
GBSpoly: Genotyping-by-sequencing protocol
GO: Gene ontology
GWAS: Genome wide association study
HCl: Hygrochloric acid
HI: Harvest index
HT: Harvest time
IAA: Indole acetic acid
IGSS: Integrated genotyping services and support
indel: Insertion-deletion
LD: Linkage disequilibrium
m.a.s.l: Meters above sea level
MAF: Minor allele frequency
MLM: Mixed linear model
mRNA: Messenger ribonucleic acid
NaCl: Sodium chloride
NaCRRI: National Crops Resources Research Institute
NaOAC: Sodium acetate
NaSARRI: National Semi-arid Resources Research Institute
NCBI: National Center for Biotechnology Information
PV: *P*-value
QQ: Quantile-quantile
QTL: Quantitative trait loci
QTN: Quantitative trait nucleotide
SNP: Single nucleotide polymorphism
SRN: Storage root number
SRY: Storage root yield
SSA: Sub-Saharan Africa
VY: Vine yield
ZFP: Zinc finger protein
